# Influence of Conductive additives on the stability of red phosphorus-carbon anodes for sodium-ion batteries

**DOI:** 10.1038/s41598-018-36797-z

**Published:** 2019-01-30

**Authors:** Rui Wang, Hanxiao Mo, Shuai Li, Yansheng Gong, Beibei He, Huanwen Wang

**Affiliations:** 1 Faculty of Materials Science and Chemistry, China University of Geosciences, Wuhan, 430074 China; 2 Academy for Advanced Interdisciplinary Studies, Southern University of Science and Technology, Shenzhen, 518055 China

## Abstract

In this paper, the influences of conductive carbons on the red phosphorus (P) composites in sodium-ion batteries are studied. Electrochemical testing results show that Ketjen Black makes the P composites present much better cycling performances. Electrochemical impedance spectra (EIS) results indicate that when Ketjen Black is used, the total resistance of the electrode can be decreased. Since Ketjen Black is a low-cost and commercially available material, our results suggest that Ketjen Black might be a promising conductor for the alloying anodes such as P in sodium-ion batteries.

## Introduction

Rechargeable lithium-ion batteries (LIBs) have been widely applied in portable electronic devices due to their excellent performances^1–4^. However, the shortage and uneven distribution of lithium resources may restrict their applications in large scale storage systems. On the other hand, Na resources are more abundant and Na has the similar chemical properties with Li. Sodium-ion batteries (SIBs) have been considered as an important alternative choice to LIBs^5,6^. However, since sodium ion (1.02 Å) is 55% larger than lithium ion (0.69 Å), it is more difficult to accommodate sodium ions reversibility in anodes. And the graphite materials commercially used as anodes in LIBs could not achieve acceptable performances in SIBs^7,8^. As a result, various materials are still being researched as anode materials for SIBs at present.

Red phosphorus (P) is one of the most promising candidates, which could react with Na to form a Na_3_P compound, leading to an extremely high theoretical capacity of 2596 mAh g^−1^ ^9–11^. Moreover, it has a low operating potential of 0.45 V (vs Na^+^/Na) and could achieve high energy densities when used in full cells. However, the successful applications of red P are hindered by two major bottlenecks: (1) pristine red P has a very low electrical conductivity (~10^−14^ S cm^−1^); (2) the large volume expansion during sodiation/desodiation may cause severe capacity decay^12–15^. Introducing carbonaceous components to synthesis composites can effectively overcome these drawbacks. Qian *et al*. reported a high energy ball-milled composite of commercial red P and carbon black (Super P), and this material could deliver a reversible capacity of 1764 mAh g^−1^ with high initial coulombic efficiency of 87%^14^. Li and co-workers used a vaporization-condensation-conversion process to synthesize P@CMK-3 and the composite shows a high reversible capacity of 1020 mAh g^−1^, with excellent rate performance and significantly enhanced cycle life^16^. Wang *et al*. reported a high pressure assisted spraying method to synthesize an unique sandwich-like P@GS composite, which presents an initial discharge capacity of 1876 mAh g^−1^ and capacity retention of 52.8% after 50 cycles^17^. In these composites, carbonaceous materials not only improve the electrical conductivity, but also serve as substrates to buffer the volume expansion of red P. And these two effects make the composites present better electrochemical performances.

In batteries, the conductor is also a vital component, especially for the materials with poor conductivity such as red P, for it determines the electronic conductivity of electrodes. Different conductors may provide totally different electrochemical performances^6,7^. Nitta *et al*. have researched the effects of conductive carbon on the stability of phosphorus anodes for lithium-ion batteries^18^. In this work, we systematically studied the influences of conductive carbons on the red P composites in sodium-ion batteries.

## Experimental

### Preparation

The red P/Super P composite was synthesized by high-energy mechanical milling (HEMM) under an argon atmosphere with a Fritsh Pulverisette 6 classic line planetary mono mill. The weight ratio of red P powder (Aladdin, 98.5%) and Super P carbon (TIMICAL Graphite & Carbon Super P) was set to 7:3, according to a previously reported work^9,19^. The starting materials were sealed in a zirconium chamber and rotated at a speed of 400 rpm for 12 h with 10 mm milling balls.

### Materials characterization

The crystalline structures of the samples were characterized by X-Ray diffraction (XRD) on a Bruker AXS D8-Focus X-ray diffractometer with Cu-Kα (λ = 1.5405 Å) radiation. Morphologies of the samples were observed by scanning electron microscopy (SEM) with a Hitachi SU3500 scanning electron microscope. Brunauer-Emmett-Teller (BET) method (Micrometrics, ASAP 2460) was used to investigate the surface areas of the 4 kinds of conductors and the pore size distributions were determined by the Barret-Joyner-Halenda (BJH) method^20,21^.

### Electrochemical characterization

The four types of conductors studied in this manuscript are Super P (Timical, batch number: 0011512), C45 (Timical, batch number: 0011511), Ketjen Black (KB, Lion, EC-600JD) and carbon nanotube (CNT, Aladdin, C139878). To make electrodes, as prepared composites were mixed with each conductor and polyvinylidene fluoride (PVDF) at a weight ratio of 8:1:1. N-Methyl pyrrolidone (NMP) was added in and the slurry is then spread on a copper foil substrate and dried in vacuum oven under 80 °C overnight. The four electrodes with different conductors were noted as PC-SP, PC-KB, PC-C45 and PC-CNT. Electrochemical performances were evaluated using 2032 coin cells with a Na plate as both counter and reference electrodes. Electrolyte was 1.0 M NaClO_4_ in polycarbonate (PC) solution with a addition of 5% (wt) fluoroethylene carbonate (FEC). The galvanostatic charge/discharge tests were tested at room temperature on a LAND battery testing system (Wuhan LAND Electronics Co., Ltd., China) within the voltage range of 0.01–2.0 V. Electrochemical impedance spectra (EIS) were recorded on a Gmary Reference 3000 unit with amplitude of 5 mV and frequency ranging from 10^6^ to 0.01 Hz. EIS tests were performed on a fresh cell, the same cell after 1 cycle, and after 20 cycles, respectively.

## Results and Discussion

**Figure 1:**
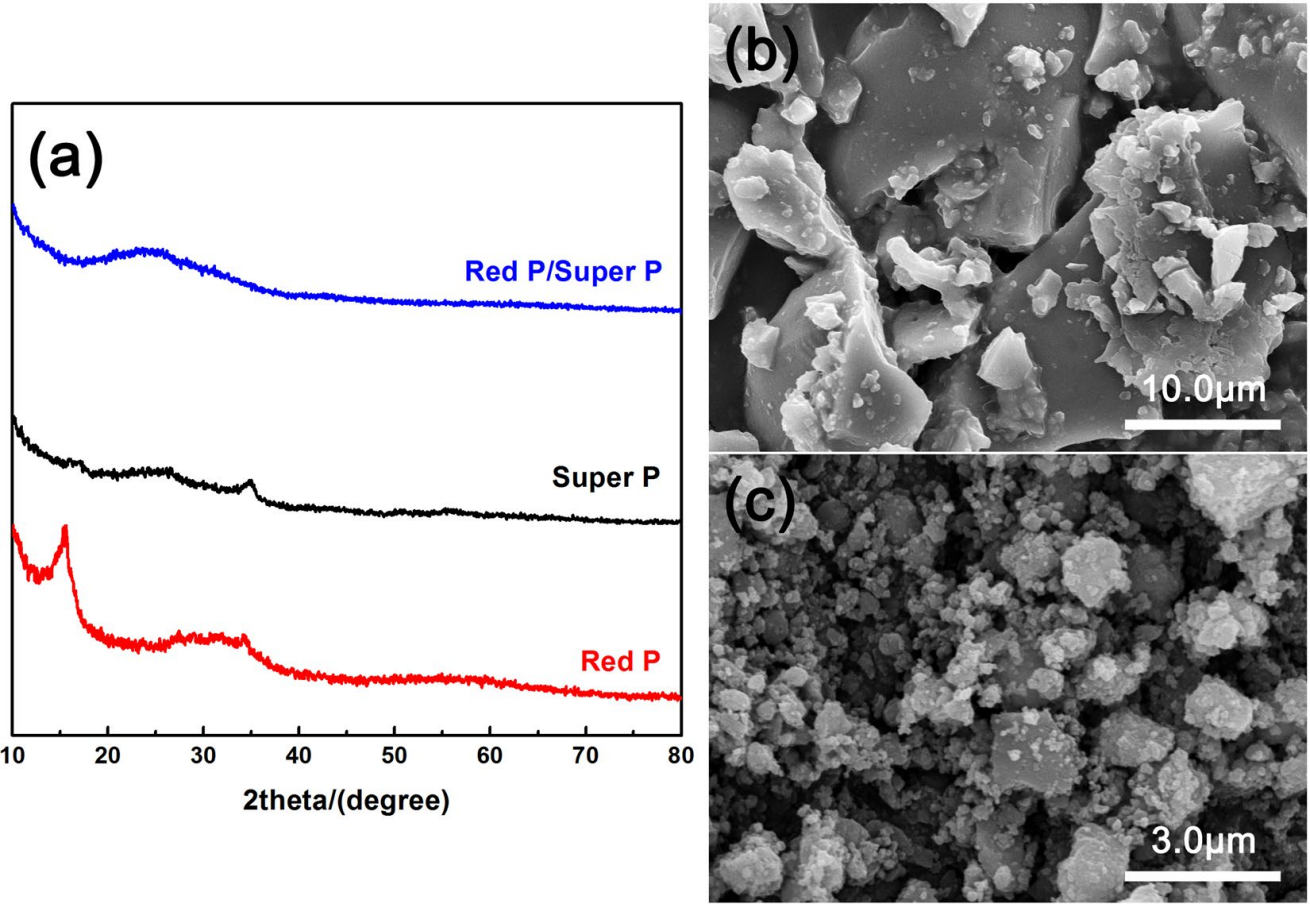
(**a**) XRD patterns of bare red P, bare Super P and red P/Super P composite; SEM images of (**b**) commercially red P and (**c**) ball-milled red P/Super P composite.

Figure 1a shows the XRD patterns of red P, Super P and their composites after ball-mill, respectively. For commercial red P, there are one peak at around 15° and a broaden bump at around 32°, which is consistent with previous reports^6^. While for Super P, there are one strong peak centered at around 34° and a bump at around 25°. After 12 h ball-milling process, red P/Super P composite only has one broaden bump at around 25°, indicating the long range ordering in red P and Super P are destroyed and the composite becomes amorphous during the long and vigorous ball-milling process. Figure 1(b) shows the SEM image of commercially red P powder, which has particle sizes of tens of micrometers. After the HEMM process, particle sizes of obtained red P/Super P composite powder decrease to several hundreds of nanometers, with agglomeration.

**Figure 2:**
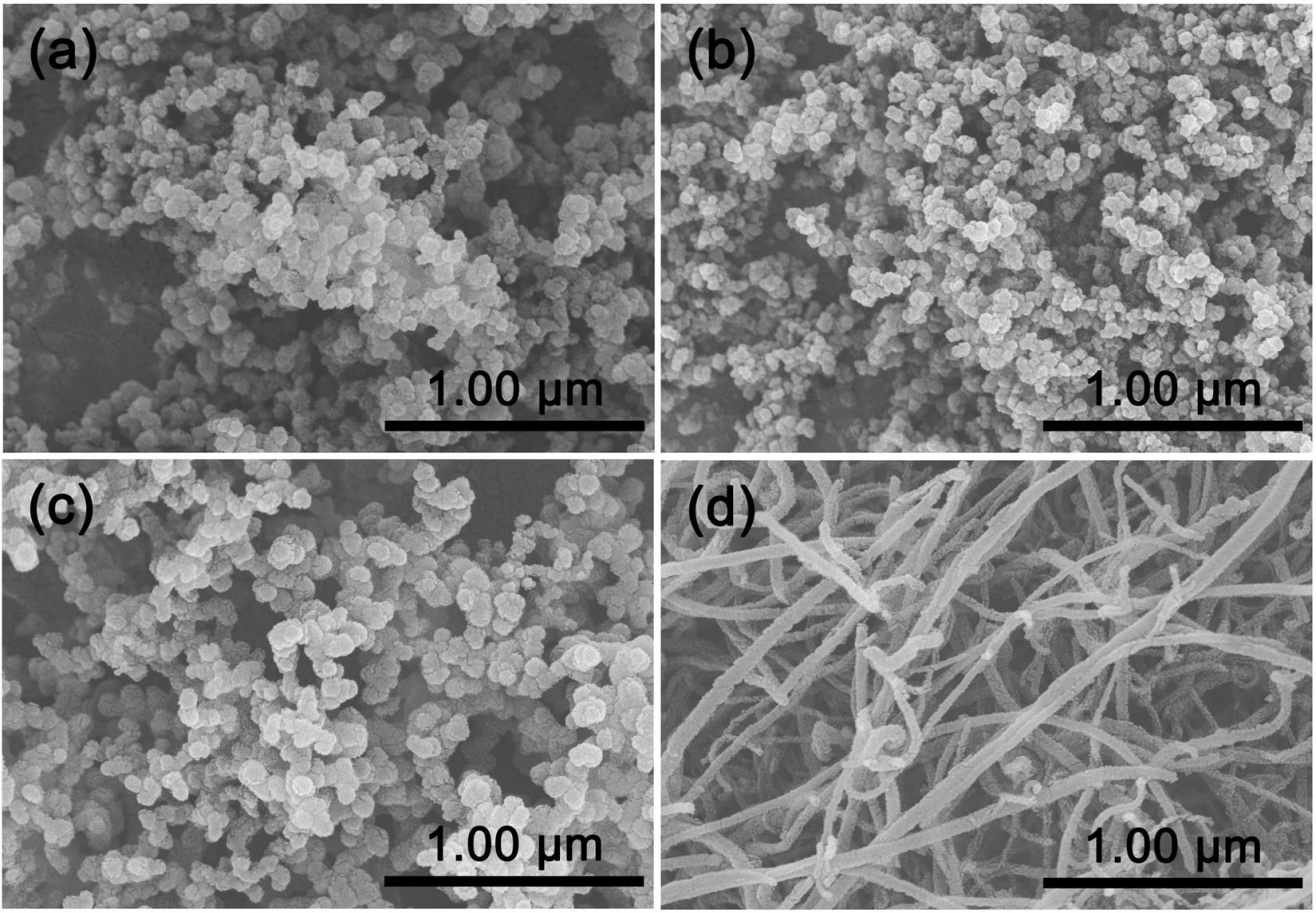
SEM images of Super P (**a**), KB (**b**), C45 (**c**) and CNT (**d**).

The SEM images in Fig. 2 reveal morphologies of the four conductors. SP and KB both present uniform particle sizes within the range of 50–100 nm and suffer a little agglomeration. Particle sizes of sample C45 are in the range of 100–200 nm, which are slightly larger than the above-mentioned two samples. Figure 2d shows the morphology of CNT, which presents tube-like shape with a diameter of about 50 nm and a length of about 100 nm.

**Figure 3:**
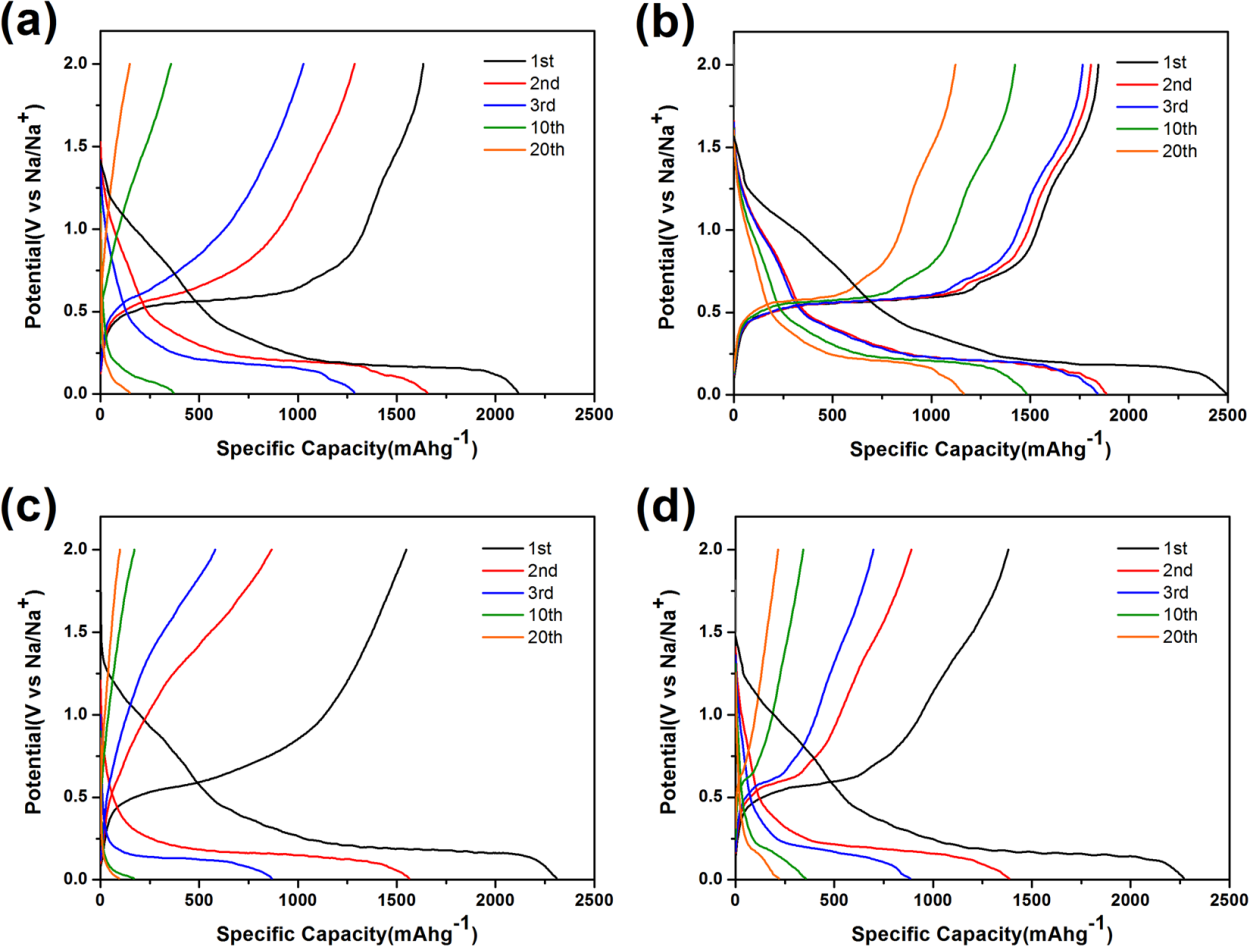
Charge-discharge curves at 200 mA g^−1^ of PC-SP (**a**), PC-KB (**b**), PC-C45 (**c**), PC-CNT (**d**).

**Figure 4:**
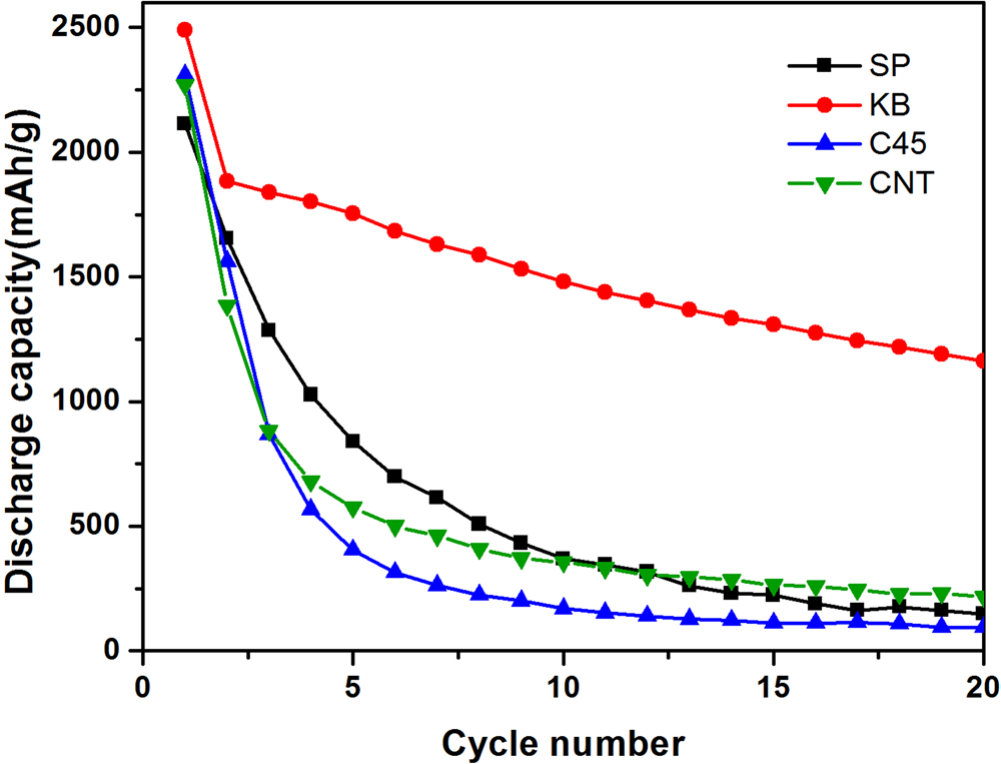
Cycle performances of PC-SP (**a**), PC-KB (**b**), PC-C45 (**c**), PC-CNT (**d**) at 200 mA g^−1^.

Figure 3 presents the charge/discharge curves of the cells with different conductors in the voltage range between 0 and 2 V at a current density of 200 mA g^−1^. All of the specific capacities are calculated based on the mass of phosphorus. It can be seen that PC-KB sample delivers an initial discharge capacity of 2490 mAh g^−1^ and a capacity of 1482 mAh g^−1^ in the 10th cycle. For other three samples, they also have high initial discharge capacities of 2114 mAh g^−1^ (PC-SP), 2307 mAh g^−1^ (PC-C45), 2269 mAh g^−1^ (PC-CNT), respectively. But after several cycles, a serious capacity fading could be observed based on the results shown in Fig. 4. It is obvious that the PC-KB sample shows much higher capacity retention (46.7%), while the other three samples only show capacity retentions lower than 10%. This result suggests that the addition of KB conductor substantially enhanced the cycling performances of red P/Super P electrode.

**Figure 5:**
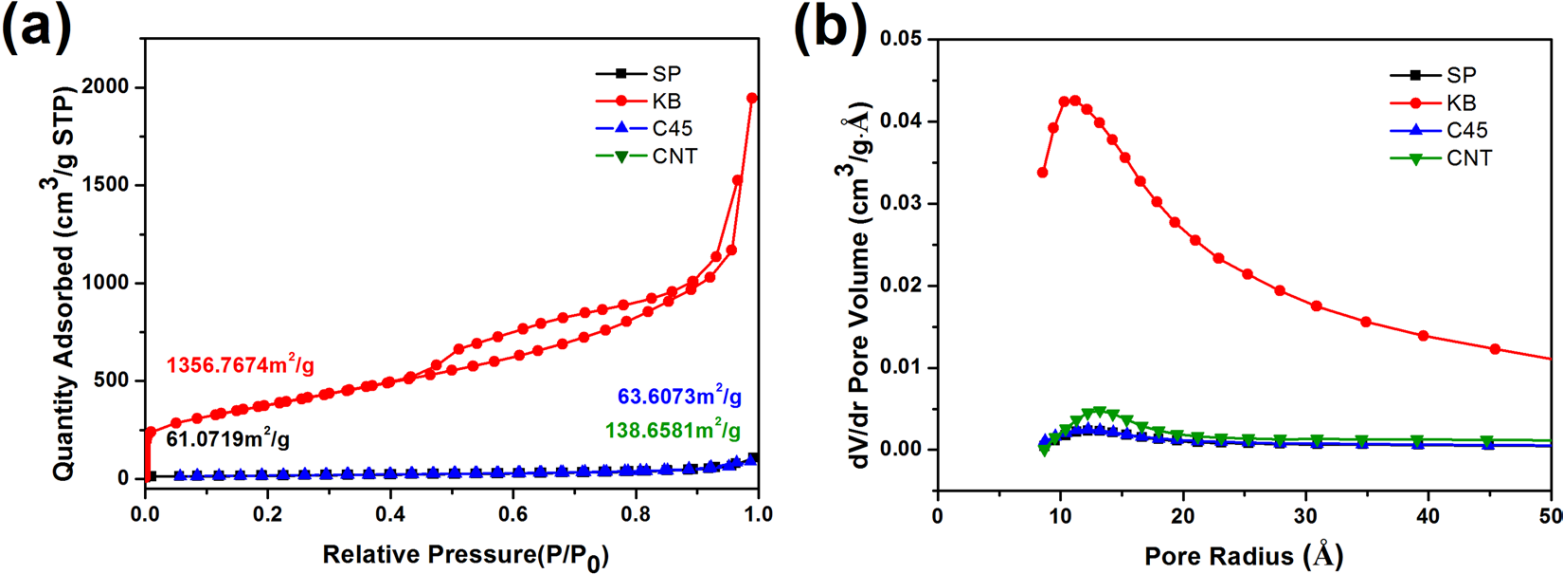
N_2_ isotherm of the four conductive powders; (**b**) Pore size distributions determined by BJH method.

In order to further investigate the reason why KB improves electrochemical performances more effectively than the other three conductors, we have done more characterizations of the conductive powders and composite electrodes. The nitrogen adsorption/desorption isotherms of four conductors are shown in Fig. 5a. The isotherm of KB exhibits typical characteristics of micro-porosity. KB has a high BET surface area of 1356.77 m^2^ g^−1^, which is about 20 times higher than SP (64.07 m^2^ g^−1^), C45 (63.61 m^2^ g^−1^), and CNT (138.6581 m^2^ g^−1^). Besides, the pore volume of KB is up to 1.64 cm^3^ g^−1^, which is also much higher than SP (0.09 cm^3^ g^−1^), C45 (0.09 cm^3^ g^−1^), and CNT (0.24 cm^3^ g^−1^). The average pore sizes of all the four samples are larger than 1.1 nm, and these pores are large enough for propylene carbonate to immerse in^22^. Since the pore volume of KB is much larger than other three samples, it might be well immersed by the electrolyte, becomes a robust particle, and acts as a buffer during the electrochemical cycles.

**Figure 6:**
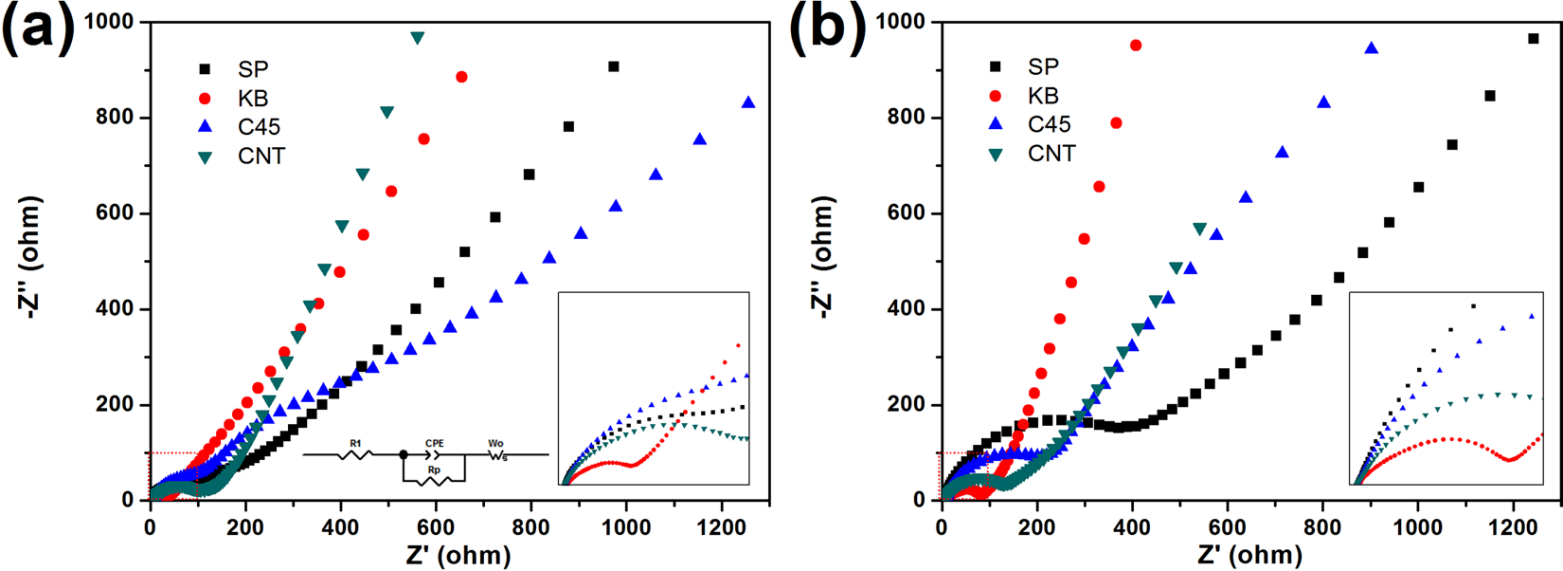
EIS curves of the four electrodes with different conductive powders after 1st cycle (**a**) and 10 cycles (**b**).

In order to further investigate the reason why KB improves electrochemical performances much more effectively than the other three conductors, electrochemical impedance spectroscopy was carried out on the cells after first cycle and after 20 cycles, respectively. The equivalent circuit for fitting Nyquist plots was shown in Fig. 6a. After first cycle, the charge transfer resistances for the PC-SP, PC-KB, PC-C45, PC-CNT samples are 114.1, 45.32, 152.4, and 106.7 Ω, respectively. It could be seen clearly that the PC-KB sample has the lowest resistance. After 20 cycles, the charge transfer resistances of all the four samples have increased, which are 496.4, 90.47, 295.9 and 155.3 Ω, respectively. The PC-KB sample still has the lowest resistance. These results indicate that when KB is used as a conductor, the total resistance of the electrode is much smaller, and this smaller resistance can keep at least for 20 cycles. In previous literatures, KB is reported to be commercially available, with a relative low price^23,24^. Our results infer that KB might be a promising conductor in the applications of alloying anode in sodium-ion batteries.

## Conclusions

In summary, we systematically studied the influences of conductive carbons on the red P composites in sodium-ion batteries. Our results show that the conductive carbons play crucial roles in the electrochemical performances of the red P composites. When KB is used as the conductor, the total resistance of the electrode can be decreased. Besides, KB might also buffer the enormous stresses from the volume expansion of P particles, alleviate the pulverization of particles and achieve better cycling performances. Our results suggest that KB might be a promising conductor in the applications of alloying anode in sodium-ion batteries.
